# Screening for type 1 diabetes: are we nearly there yet?

**DOI:** 10.1007/s00125-018-4774-0

**Published:** 2018-11-13

**Authors:** Parth Narendran

**Affiliations:** 1 Institute of Immunology and Immunotherapy, College of Medical and Dental Sciences, University of Birmingham, Edgbaston, Birmingham, B15 2TT UK; 2 Department of Diabetes, University Hospitals Birmingham NHS Foundation Trust, Birmingham, UK

**Keywords:** Diabetic ketoacidosis, Screening, Type 1 diabetes mellitus

On the 31 December 2014, 13-year-old Peter Baldwin from Cardiff, UK, was taken to his GP with symptoms of a worsening chest infection, thirst and tiredness. His blood glucose was not checked, new-onset type 1 diabetes was missed, and Peter subsequently went on to develop severe diabetic ketoacidosis (DKA). Despite the best efforts of paramedics and healthcare staff, Peter unfortunately did not get past the first few days of his type 1 diabetes and passed away soon after. A tragic loss to all that knew him, and of a promising young life [1].

Unacceptably high proportions of children are diagnosed in DKA (24% in England and Wales, 30% in the US state of Colorado [2, 3]). In this issue of *Diabetologia*, Lundgren and colleagues report the results of a study that extends our understanding of some of the benefits of screening and allows us to explore more proactive approaches to diagnosing type 1 diabetes [4].

Detectable evidence of beta cell immunity precedes the clinical presentation of type 1 diabetes by a number of years [5]. This immunity is most efficiently measured by a blood test for autoantibodies to beta cell-associated proteins such as GAD, insulin and islet antigen 2 (IA2) [6]. Combined with the use of genetic markers [7] and oral glucose challenge, these tests allow detection of people at risk of developing type 1 diabetes long before the onset of hyperglycaemia and of symptoms [8]. Currently, these investigations are used for research purposes, to understand the natural history and development of type 1 diabetes, and to identify individuals in whom prevention therapies can be tested.

However, cases like those of Peter Baldwin, and the significant proportion of children who present with DKA at diagnosis, make us question whether these screening tests should be rolled out to the general population. This is an emotive topic and one we should explore carefully. The current thinking on this subject suggests we should *not* be routinely screening for type 1 diabetes because there is no therapy currently proven to prevent or significantly delay the onset of this condition. This is supported by guidelines from respected authorities in this area [9]. However, this does not consider the benefits associated with early detection of type 1 diabetes, not least of which is prevention of death by DKA.

So, what are the benefits of screening for type 1 diabetes? A number of research studies have now outlined what these may be (Table 1). These research studies have either screened the general population or been more focused and screened people deemed at risk because they have a family member with the disease. Screening has been undertaken through genetic and autoantibody tests followed by glucose tolerance tests for those deemed at risk. To date, screening studies have largely studied children and not explored adults (the age group in which almost 40% of type 1 diabetes presents). The benefit most consistently reported across these studies is avoidance of DKA. This is a significant benefit, with a reduction from a quarter of new type 1 diabetes being diagnosed in DKA down to 3%. Also, presumably because they are diagnosed at an earlier stage of disease, there is more residual beta cell function (as measured by C-peptide), lower insulin requirements and lower HbA_1c_ at the time of diagnosis [10–15]. The paper by Lundgren and colleagues [4] adds to the evidence. They report from follow-up of the Diabetes Prediction in Skane (DiPiS) study, where almost 40,000 children in southern Sweden were screened for type 1 diabetes. Of these, 6000 were deemed to be at some degree of risk and offered follow-up. About two-thirds accepted this offer of follow-up. Lundgren and colleagues report on the outcome of the 51 children who developed type 1 diabetes in the follow-up group, compared with the 78 who developed type 1 diabetes but had not accepted the offer of follow-up. Children who chose follow-up had a lower frequency of DKA (2% vs 18%), and lower HbA_1c_ (9 mmol/mol [0.8%] lower) at diagnosis. Importantly, HbA_1c_ remained significantly better up to 5 years after diabetes diagnosis. A potential caveat is that a greater proportion of the participants who opted for follow-up had mothers of Swedish origin, and a greater engagement in research may reflect a propensity for greater involvement in diabetes care following diagnosis. That said, this is the first time that such a prolonged HbA_1c_ benefit has been reported, and this HbA_1c_ benefit has a clear clinical and economic benefit. Furthermore, since HbA_1c_ generally tends to rise in the first years after diagnosis, and then stabilise and ‘track’ after about 5 years [16], we could postulate that the lower HbA_1c_ levels in the follow-up group persist over the long term.

**Table 1:** Benefits associated with screen-detected type 1 diabetes

Study	Age group	Less DKA	Lower HbA_1c_	Lower insulin dose	Shorter period in hospital	Others
BabyDiab and Munich Family study [27]	Paediatric	Y	Y	N	Y	
DiPiS [10, 11]	Paediatric	Y	Y	N	ND	
TEDDY [13]	Paediatric	Y	Y	Y	ND	Higher residual C-peptide
DAISY [12]	Paediatric	Y	Y	Y	Y	
DIPP [14, 15]	Paediatric	Y	Y	ND	ND	Less weight loss

DAISY, Diabetes Autoimmunity Study in the Young

DiPiS, Diabetes Prediction in Skane

DIPP, Finnish Type 1 diabetes Prediction and Prevention

ND, not determined

TEDDY, The Environmental Determinants of Diabetes in the Young

Do these benefits make a workable case for screening? There are those who would put forward a scientific argument to support this case [17, 18]. To provide some clarity, Table 2 presents the benefits of screening against the WHO guidelines for screening, originally proposed almost 50 years ago [19]. Here, the benefits relate to early detection, and *not* to prevention of the disease. There are a number of criteria that remain to be satisfied.

**Table 2:** Screening for type 1 diabetes set against WHO criteria for screening [19]

Criterion	Satisfied
The condition sought should be an important health problem	Yes Type 1 diabetes is an important health problem. Whilst early screening does not currently allow us to institute preventative therapy, it may prevent comorbidity associated with late presentation
There should be an accepted treatment for patients with recognised disease	Yes People at risk will be provided with education until they are formally diagnosed with diabetes, at which time they will be initiated on insulin. Early education and initiation of insulin are likely to be acceptable and effective
Facilities for diagnosis and treatment should be available	Yes Most healthcare facilities have access to phlebotomy and oral glucose challenge facilities. Samples can be sent to reference centres nationally for analysis
There should be a recognisable latent or early symptomatic stage	Yes Latent and early symptomatic phase can be detected through autoantibody and glucose challenge
There should be a suitable test or examination	Yes Peripheral blood tests for antibodies and oral glucose challenge
The test should be acceptable to the population	Not known The psychological consequences of awareness of high risk of a chronic disease for which there is no cure is not known
The natural history of the condition, including development from latent to declared disease, should be adequately understood.	Not known Natural history remains to be fully elucidated, different rates of progression remain to be understood. Age, ethnicity and environment appear to influence natural history and these effects remain to be elucidated
There should be an agreed policy on whom to treat as patients	Yes People fulfilling standard WHO criteria for diabetes will be treated as diabetic
The cost of case-finding should be economically balanced in relation to possible expenditure on medical care as a whole	Not known
Case-finding should be a continuing process and not a ‘once and for all’ project	Yes A long-term programme can be implemented nationally

First, we do not know the psychological consequences of alerting a person to a condition for which there is no current cure. Granted, this time may usefully be spent in educating and preparing the person for managing type 1 diabetes. However, concurrent work on the psychological impact of informing patients of high risk of type 2 diabetes suggests that, even with a condition that can be significantly delayed, there can be a negative psychological impact. These include negative markers of mental health, reduced motivation and lack of engagement with behaviour change [20].

Second, the natural history of type 1 diabetes is still not clearly understood, and the influence of age, ethnicity and environment remain to be elucidated. The environment may influence the rate of development, as evidenced by migration studies, where populations migrating from areas of low incidence to high appear to adopt the risk of the host population [21]. Whilst we previously believed that rates of beta cell loss were faster at a younger age, more recent work suggests that the rate may remain the same across the age spectrum [22], despite islet histology changing with age of presentation [23]. Importantly, all major screening studies to date have focused on children.

Lastly, the cost benefit needs to be established for a formal screening programme. Since 2015 the Bavarian Fr1da study has been screening children aged 3–4 years for type 1 diabetes [24]. The aim is to screen 200,000 children, with each screening roughly costing 20 Euros per child. If DKA and hospitalisation is prevented in 200 children, this cost saving will in itself cover a third of the cost of the study. Furthermore, patients presenting with DKA tend to have an HbA_1c_ that is up to 1.4% higher than those who do not over the long term [3]. If the lower HbA_1c_ reported by Lundgren et al persists to reduce the incidence and economic impact of diabetic complications, combined with the saving on DKA cost, we may be a significant way to covering the economic cost of screening. Further work is required in this area.

Until some of the issues above are resolved (and there is significant work ongoing) the way forward is in public and healthcare education, and raising awareness. As a direct result of the efforts of Peter Baldwin’s family, the Welsh government recently (October 2018) discussed ten recommendations around raising awareness of type 1 diabetes. These recommendations include adopting the Diabetes UK 4Ts campaign [25], and a recommendation that all cases diagnosed in DKA are reviewed for shared learning [26]. These measures are critical if we are going to make a meaningful change to the devastation caused by death by DKA.

## Abbreviation

DKA: Diabetic ketoacidosis
